# The Effect of Bacterial Signal Indole on the Electrical Properties of Lipid Membranes

**DOI:** 10.1002/cphc.201200793

**Published:** 2013-01-09

**Authors:** Catalin Chimerel, Andrew J Murray, Enno R Oldewurtel, David K Summers, Ulrich F Keyser

**Affiliations:** [a] Cavendish Laboratory, Department of PhysicsUniversity of Cambridge, JJ Thomson Avenue, Cambridge, CB3 0HE (United Kingdom), Fax: (+44) (0)1223 337000; [b] Department of Physiology, Development & NeuroscienceUniversity of Cambridge, Downing Street, Cambridge, CB2 3EG (United Kingdom); [c] Department of GeneticsUniversity of Cambridge, Downing Street, Cambridge, CB2 3EH (United Kingdom)

**Keywords:** bilayers, electrophysiology, ionophores, lipids, membranes

## Abstract

Indole is an important biological signalling molecule produced by many Gram positive and Gram negative bacterial species, including *Escherichia coli*. Here we study the effect of indole on the electrical properties of lipid membranes. Using electrophysiology, we show that two indole molecules act cooperatively to transport charge across the hydrophobic core of the lipid membrane. To enhance charge transport, induced by indole across the lipid membrane, we use an indole derivative, 4 fluoro-indole. We demonstrate parallels between charge transport through artificial lipid membranes and the function of complex eukaryotic membrane systems by showing that physiological indole concentrations increase the rate of mitochondrial oxygen consumption. Our data provide a biophysical explanation for how indole may link the metabolism of bacterial and eukaryotic cells.

## 1. Introduction

Indole is an important biological signal molecule produced by more than 85 Gram positive and Gram negative bacterial species.[1] In bacterial communities, indole acts as an inter- and intra-cellular signal, influencing multiple aspects of bacterial physiology and has proved to be an important factor in the transition to stationary phase.[2,3] It also promotes resistance to a range of drugs and toxins[4] and is involved in preventing plasmid loss.[5] Aspects of bacterial ecology and host–pathogen interactions that respond to indole include biofilm formation[6] and the expression of virulence factors.[7] In addition to its multiple functions as bacterial signal, recent evidence suggests that indole has a role in communication between intestinal bacteria and their mammalian hosts.[8,9] This suggests that indole acts as an inter-kingdom signal,[8,9] an activity facilitated by its ability to pass freely through lipid membranes.[10] However, the biophysical effects of indole used by bacteria to communicate with eukaryotic cells remain largely unclear.

Some 30 years ago a study suggested that indole influences oxidative phosphorylation in rat liver mitochondria.[11] According to Mitchell**’s** chemiosmotic hypothesis,[12,13] the generation of the key energy storage molecule, ATP, by oxidation and phosphorylation relies on the ability of the lipid membrane to withstand an electrochemical proton gradient. Experiments with artificial lipid membranes have shown that weak acids[14] act as proton ionophores (protonophores), by passively transporting protons across lipid membranes. In essence, protonophores uncouple oxidation and phosphorylation by providing a shunt for the transport of protons across the inner membrane of bacteria and mitochondria of eukaryotes. Consistent with the reported effect of indole on mitochondria, we have recently proved that indole concentrations in the mm range stop bacterial cell division by dissipating the membrane potential across the inner membrane.[15]

Despite the importance of indole as a signalling molecule and the evidence that it functions as an ionophore, there has been no detailed investigation of the capacity of indole to transport charge across lipid membranes. Here, we analyse the biophysical effect of indole on the passive diffusion of ions through artificial lipid membranes. We demonstrate that an indole derivative, 4 fluoro-indole (4 F-indole), transports charge more than one order of magnitude faster across lipid membranes. Further, we link the effects of charge transport through artificial lipid membranes with a biological system by showing that physiological concentrations of indole increase the rate of mitochondrial oxygen consumption and thus are likely to dissipate the proton motive force in eukaryotic cells.

## 2. Results and Discussion

### 2.1. Theoretical Background

The transport of charge through artificial lipid membranes has been studied for a great variety of uncoupling agents.[19] Among these we differentiate the heterocyclic aromatic compounds which act as protonophores: benzimidazoles.[19–24] It can be observed that the chemical structure of indole is similar to the chemical structure of benzimidazoles. In consequence, we expect that indole and its derivative 4 F-indole will have a charge transport mechanism similar to benzimidazoles. The equilibrium theory described here[25,26] has been previously developed and successfully applied[14,20–23,26] for molecules, such as 4,5,6,7-tetrachloro-2-trifluoromethyl benzimidazole (TTFB) and 5,6-dichloro-2-trifluoromethyl benzimidazole (DTFB). Therefore, we apply the same theoretical model to indoles.

This theoretical model[25,26] proposes that the mechanism of charge transport involves the formation of a dimer between the uncharged form [HA] and the ionized form [A^−^]. The dimer [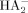] has lower solvation energy for passing through the hydrophobic core of the lipid bilayer as compared to the ionized form [A^−^], the hydrogen ion or the hydroxide ion. If the charged dimer is the only species shuttling charge across the hydrophobic barrier posed by the lipids, the induced membrane conductance (*G*) is proportional to the concentration of dimer [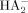] and it can be expressed as shown in Equation (1):[25,26]
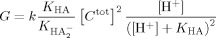
(1)where *k* is a constant proportional to the product of the mobility of 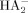 in the membrane and the partition coefficient of 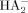 between water and the membrane, 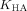 is the equilibrium constant for the dissociation of the HA molecule into H^*+*^ and A^*−*^, 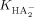 is the equilibrium constant for the dissociation of 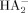 into HA and H^+^, [*C*^tot^] is the total uncoupler concentration.

Experimentally a small ionic current is found to pass directly through the lipid membrane in the absence of the uncoupler molecules. Consequently the measured membrane conductance (*G*_m_) is the sum of the uncoupler-induced membrane conductance (*G*) plus the leakage membrane conductance (*G*_0_).

For simplicity, the dependence of uncoupler-induced membrane conductance as a function of uncoupler concentration, expressed in Equation (1), can be rewritten as Equation (2):
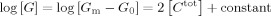
(2)

As shown by Equation (1), the model predicts that the membrane conductance increases with the square of the uncoupler concentration. This is a consequence of the cooperative action between two uncoupler molecules. If a monomolecular transport model were to be employed, then a linear increase in the membrane conductance with the uncoupler concentration would be expected.[14] In addition, Equation (1) also predicts a dependence of the conductance on the pH of the solution. The conductance is expected to increase when the pH is increased, to reach a maximum when the pH is equal to the p*K*_a_ of the uncoupler, and to decrease as the pH is further increased.

In the literature the p*K*_a_ of indole is reported with values between 16.7 and 16.9.[27,28] Therefore, the hydrogen concentration [H^+^] in the pH region from 2 to 12 (the range of our measurements) is much larger than the equilibrium constant for the deprotonation of the indole molecule (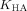). The fluoride atom present in the 4 F-indole molecule withdraws the electron density from the aromatic ring by inductive effect, producing a decrease in the proton affinity of the NH group.[29] Therefore, the p*K*_a_ of 4 F-indole is expected to be smaller than the p*K*_a_ of indole. In the literature there is no measurement for the p*K*_a_ of 4 F-indole. However, if we take into account the p*K*_a_ reported for indole molecules substituted in the same position with other electron-withdrawing groups, such as 4 NO_2_-indole (p*K*_a_=14.5) and 4 Cl-indole (p*K*_a_=15.8), we can estimate the p*K*_a_ of the 4 F-indole molecule as being in the same range.[28] Hence, we expect that our assumption, 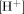 still holds for 4 F-indole and then Equation (1) can be simplified to Equation (3):
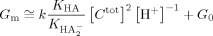
(3)

Therefore, the model predicts that the uncoupler-induced membrane conductance is inversely proportional to the hydrogen concentration present in solution when the pH is much smaller than the p*K*_a_ of the uncoupler [Eq. (3)].

At constant uncoupler concentration Equation (3) can be rewritten to Equation (4):
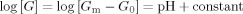
(4)

In addition, the model predicts that even though the only charge permeating through the membrane is the [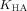] complex, the membrane behaves as if it were selectively permeable to [H^+^] ions. The Nernstian behaviour for the trans-membrane potential (*V*), required to stop the ionic current that passes through the lipid membrane when a pH difference exists between the CIS and TRANS chamber of the bilayer, is described by Equation (5):[25,26]
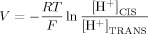
(5)

Equation (5) predicts that at 21 °C the Nernstian trans-membrane potential needed in order to stop the current flow due to a pH gradient of one unit is 58.4 mV. However, if the leakage membrane conductance is comparable to the membrane conductance induced by the uncoupler molecules, then the measured trans-membrane voltage (*V*_m_) is smaller than the Nernstian value.[30,31] In this case, the leakage conductance creates a voltage divider (see the Supporting Information Figure S3) which reduces the measured trans-membrane potential *V*_m_ by a factor depending on *G*_m_ and *G*_0_ as shown in the literature [Eq. (6)]:[30,31]
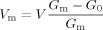
(6)

Here, the terms of the equation are the same as previously defined in Equations (1)–(3).

### 2.2. The Conductance of the Lipid Membrane in the Presence of Indole and 4 F-Indole

**Figure 1:**
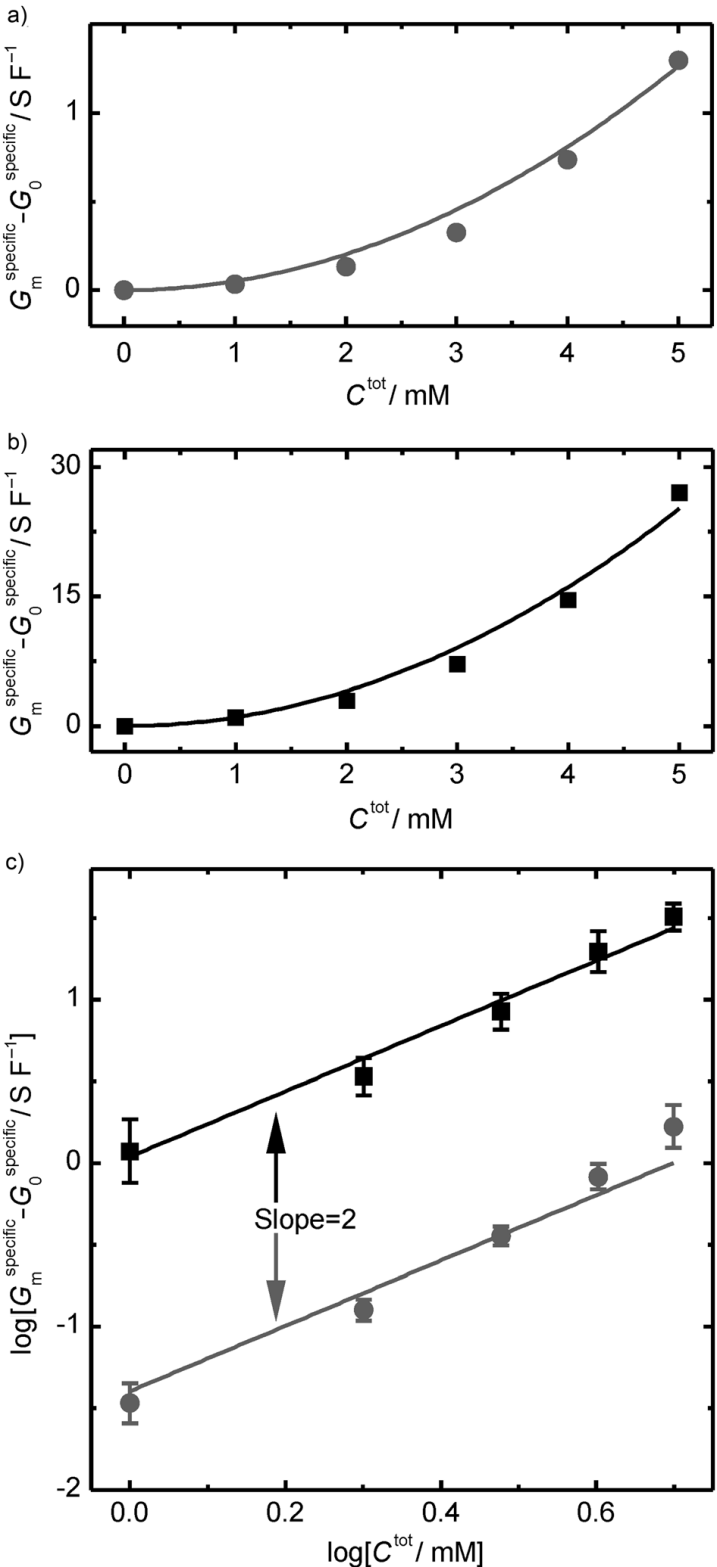
The specific conductance of the lipid membrane (aqueous solution, pH 7.5) as a function of the indole concentration (circles) and 4 F-indole concentration (squares). Specific membrane conductance plotted as a function of the a) indole concentration and b) 4 F-indole concentration. The curves through the points are fitted according to Equation (1). c) Logarithm of the bilayer conductance plotted against the logarithm of the concentration of indole or 4 F-indole (1 mm to 5 mm only). The curves through the points are drawn according to Equation (2). In panels (a) and (b) single traces are shown, while in panel (c) the means and standard deviations are calculated from five independent repeats.

First, we analyse our electrophysiology measurements which demonstrate that indole and 4 F-indole transport charge across artificial lipid membranes. A black lipid membrane (BLM) is reconstituted in a classic electrophysiology setup[16,17] and the membrane conductance is measured upon the addition of indoles. In Figure 1 a, b single representative experiments are presented. It can be observed that the specific conductance of the lipid membranes, calculated as described in the Experimental Section (*G*_m_^specific^−*G*_0_^specific^), increases with the increase in concentration (*C*^tot^), of indole or 4 F-indole. The dependence of the indole-induced specific conductance (*G*_m_^specific^−*G*_0_^specific^) on the indole or 4 F-indole concentration can be described with a quadratic function as predicted by Equation (1) (Figure 1 a, b). This supports the hypothesis that indole forms dimers when uncoupling the membrane. To complement Figure 1 a and b with a better representation of the data, the logarithm of the indole-induced specific conductance, *G*_m_^specific^−*G*_0_^specific^, is plotted as a function of the logarithm of the substrate concentration *C*^tot^ (Figure 1 c). Here, an average of five different experiments is shown for both indole and 4 F-indole. The data can be described with Equation (2). At concentrations over 3 mm the experimental data increase faster than the proposed fit (Figure 1 c). This is probably due to the additional leakage current passing through the lipid bilayer when the indole or 4 F-indole concentration is increased. In support of this hypothesis we observed that the stability of the lipid bilayer decreases when the indole concentration exceeds 5 mm.

The dependence of membrane conductance on the concentration of indole and 4 F-indole can be explained qualitatively within the framework of the theoretical model. The quadratic dependence of membrane conductance on the indole or 4 F-indole concentration is predicted by Equation (1), and is a consequence of the cooperative action between the uncoupler molecules. The increased conductance in the presence of 4 F-indole is due to the electronegative nature of the F atom, which delocalizes the charge on the indole dimer [I_2_H^−^], and thus it reduces the energy required for the deprotonated 4 F-indole to pass through the hydrophobic core of the lipid membrane.[32] As the current-versus-voltage characteristics measured for both indole and 4 F-indole are sub-linear (Figure S4), it is suggested[33] that the rate-limiting step for the conduction process is the passage of the charged complex through the lipid membrane. Consequently, 4 F-indole is a better ionophore increasing *G*_m_^specific^−*G*_0_^specific^ by a factor of ∼21 compared to indole.

### 2.3. The Selectivity of Charge Transport in the Presence of Indole and 4 F-Indole

**Figure 2:**
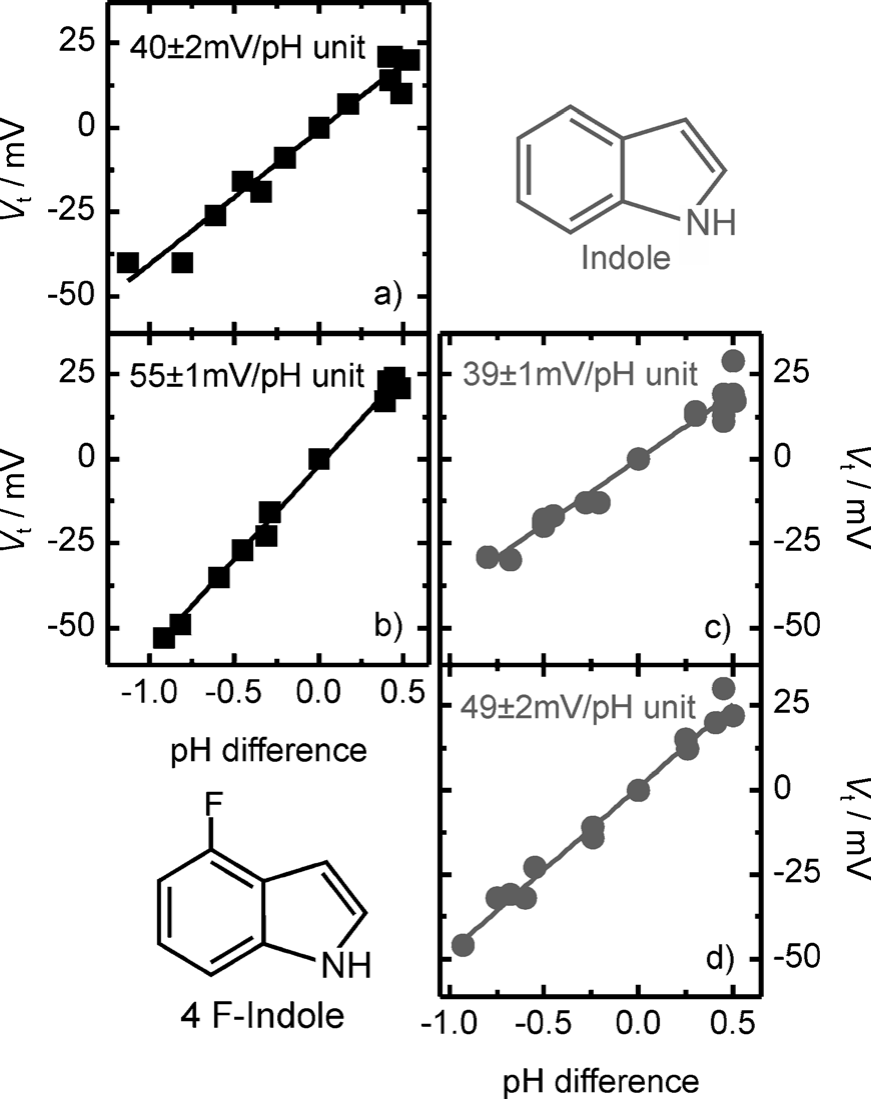
Selectivity of charge transport for indole and 4 F-indole. The dependence of the membrane potential (*V*_t_) required to stop the current generated by a pH difference between the TRANS and the CIS chambers of the lipid bilayer in the presence of a) 0.5 mm 4 F-indole, b) 2.5 mm 4 F-indole, c) 2.5 mm indole, and d) 5 mm indole. The measurements were started at pH 7 in 15 mm PB buffer and 100 mm KCl. The pH was modified in the CIS chamber by the addition of HCl or KOH in aqueous solution. For a pH lower than 7 in the CIS chamber the pH difference is denoted as positive and for a pH higher than 7 as negative. For each set of data at least four different bilayers were investigated and the raw data are presented here. A straight line was fitted through the data and the trans-membrane voltages per pH unit were found from the slope of the linear fit.

Furthermore, we tested the selectivity of charge transport for indole and 4 F-indole by investigating the response of the lipid bilayer to a pH gradient. When a pH gradient is formed across a lipid membrane in the presence of indole or 4 F-indole, an ionic current flows through the membrane, even if there is no voltage applied (Figure S4). If the pH is lower in the CIS chamber (Figure S1), an equivalent proton current is flowing from the CIS to the TRANS chamber. For both indole and 4 F-indole, the membrane potential (*V*_t_) required to stop the current generated by the pH gradient has a linear dependence on the magnitude of the pH difference between the TRANS and the CIS chambers of the lipid bilayer apparatus. In Figure 2 we show the dependence of *V*_t_ on the pH difference for two concentrations of indole (2.5 mm and 5 mm) and 4 F-indole (0.5 mm and 2.5 mm). A linear fit is applied to the data to determine the measured trans-membrane potential required to stop the current generated by a pH gradient of one pH unit (*V*_m_). For 4 F-indole we found *V*_m_ to be 40±2 mV per pH unit at 0.5 mm (Figure 2 a). Increasing the concentration to 2.5 mm leads to an increase in *V*_m_ to 55±1 mV per pH unit (Figure 2 b). For indole we find a similar trend, with 39±1 mV per pH unit obtained at 2.5 mm (Figure 2 c) and rising to 49±2 mV per pH unit at 5 mm (Figure 2 d). The trans-membrane potential measured increases towards the Nernstian prediction of 58.6 mV per pH unit, when the concentration of the indoles is increased. This effect is explained by the presence of the leakage conductance (*G*_0_ is around ∼2 pS) and was discussed earlier in Equation (6). Therefore, in the next paragraph we present the theoretical predictions obtained with Equation (6) by plugging in the experimental values measured for the leakage and conductance in the presence of indole.

The total membrane conductance *G*_m_ measured in the presence of 2.5 mm indole is 9.3±2.7 pS and in the presence of 0.5 mm 4 F-indole is 10.9±3.8 pS. Using Equation (6) we predict the measured trans-membrane voltage, *V*_m_, to be 45.8±3.6 mV per pH unit for 2.5 mm indole and 47.3±3.7 mV per pH unit for 0.5 mm 4 F-indole. These values are close to our experimental data presented in Figure 2. We suggest that the slight difference between the measured values and the theoretical predictions comes from an underestimation of the leakage conductance in the presence of indole. As mentioned earlier, a significant increase in leakage conductance at high indole concentrations is suggested by the data in Figure 1 c. At concentrations above 3 mm the increase in conductance with concentration is slightly more than the quadratic dependence. Increasing the concentration to more than 5 mm makes the lipid bilayer prone to rupture. In the case of 4 F-indole the deviation from the quadratic increase is much less than that of indole, and indeed it reflects a trans-membrane voltage closer to the Nernst potential (55±1 mV per pH unit). Hence, our data indicate that indole and 4 F-indole show characteristics typical of protonophores.

**Figure 3:**
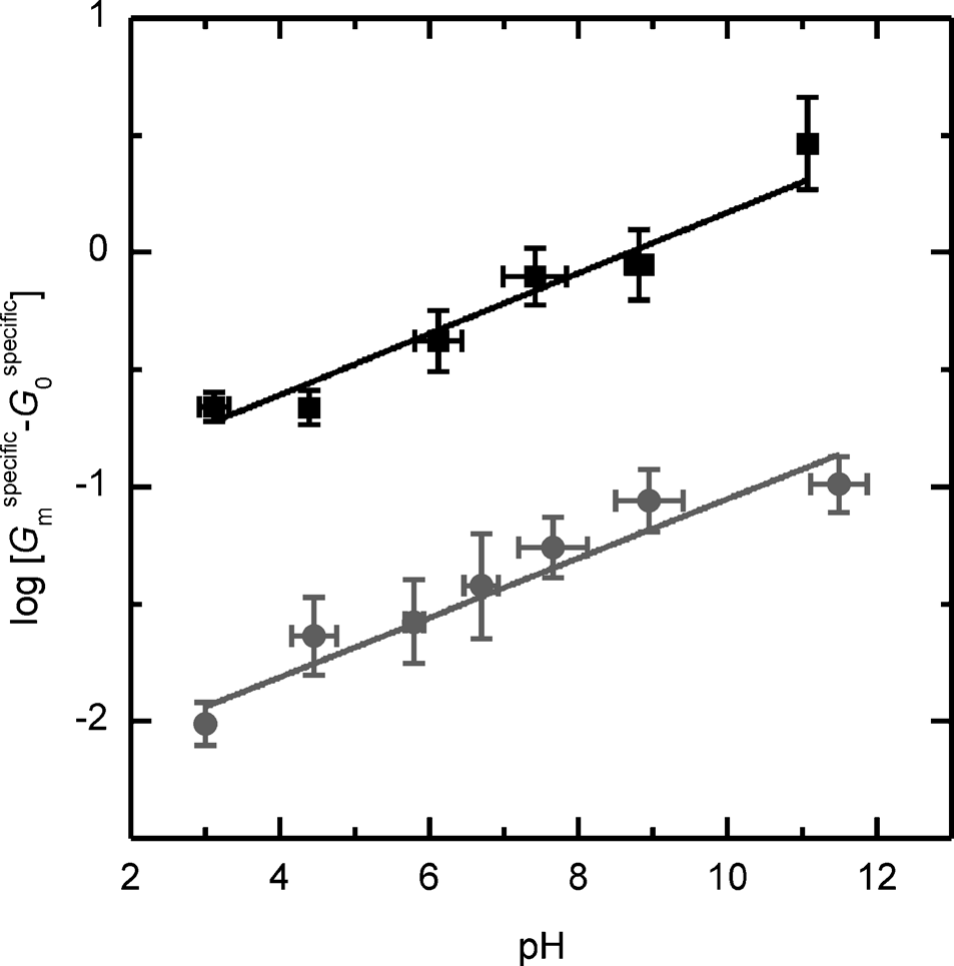
Dependence of the logarithm of the specific membrane conductance on the pH of the bathing solution in the presence of 2.5 mm indole (circles) or 2.5 mm 4 F-indole (squares). The aqueous solution contained 0.1 m KCl and 15 mm PB buffer, 15 mm potasium citrate and 15 mm tris. The pH was modified by the addition of KOH or HCl. The curves through the points is drawn according to the empirical Equation (7).

Moreover, the pH dependence of the specific membrane conductance minus the specific leakage conductance, *G*_m_^specific^−*G*_0_^specific^, in the presence of either 2.5 mm indole or 4 F-indole was measured. Increasing the pH of the solution in the presence of 2.5 mm indole or 2.5 mm 4 F-indole led to a change of ionic conductance through the lipid membrane (Figure 3). Nevertheless, the increase is slower than predicted by the theoretical model [Eq. (4)]. Consequently, we fitted the experimental data to the empirical Equation (7), which is similar to Equation (4):
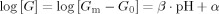
(7)where *α* and *β* are empirical fitting parameters.

The theoretical model predicts *β*=1 [Eq. (4)]. Experimentally we obtain *β*=0.127±0.014 for 2.5 mm indole and *β*=0.130±0.019 for 2.5 mm 4 F-indole. Thus, the increase in conductance with the increase in pH is less than predicted by Equation (4). This means that the membrane conductance does not strictly follow the increasing formation of ionized dimer species [A_2_H^−^] in solution. Therefore, the model fails to explain the pH dependence. In the literature a similar behaviour is observed for other ionophore molecules, for example, decylamine and picric acid.[34,35] To the best of our knowledge a satisfactory explanation for this behaviour has not yet been formulated and is beyond the scope of this paper.

### 2.4. The Effect of Indole and 4 F-Indole on the Mitochondrial Oxygen Consumption

After discussing the effects of indole and 4 F-indole we now turn to the biological relevance of these protonophores and their effect on eukaryotic mitochondria. The generation of ATP by mitochondria in eukaryotic cells depends on the establishment of a proton gradient across the inner mitochondrial membrane. This proton pumping is associated with the multi-step transfer of electrons from NADH or FADH_2_ to oxygen through the electron transport chain (ETC). If the proton gradient is reduced, for example, by an ionophore, this then allows the protons to pass through the membrane, operation of the ETC is stimulated, and oxygen consumption is increased. As we showed herein, indole and 4 F-indole are proton ionophores and therefore should stimulate mitochondrial oxygen consumption.

**Figure 4:**
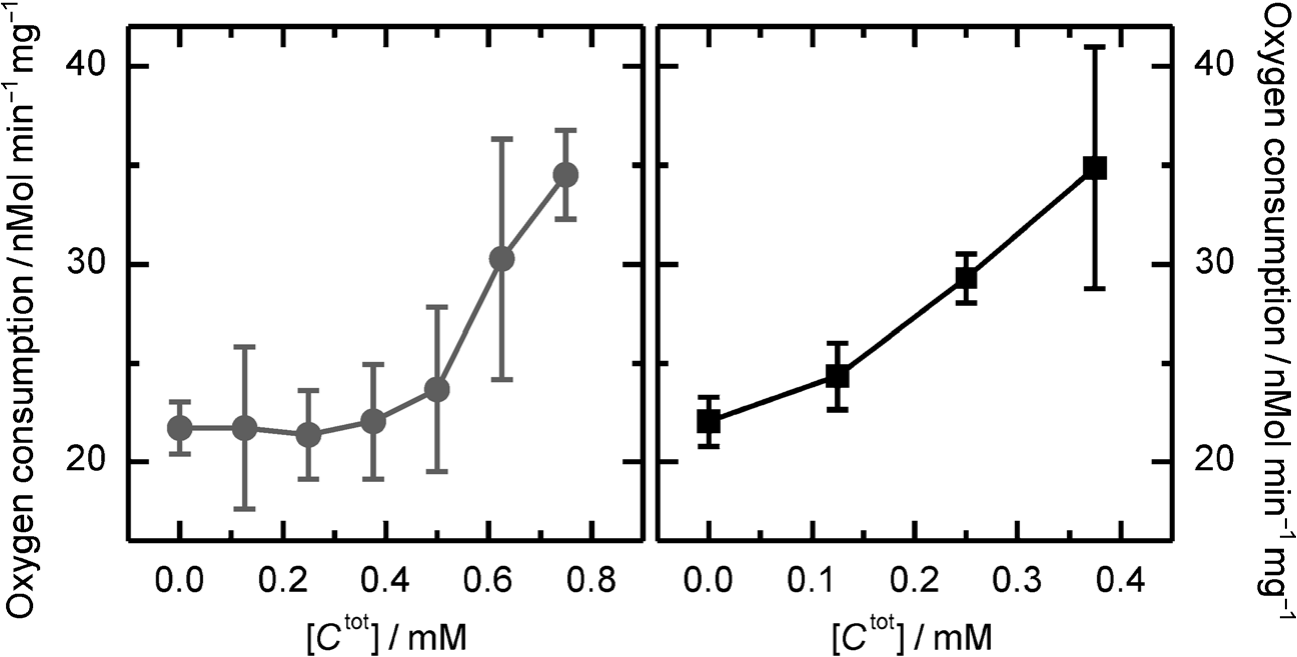
Stimulation of oxygen consumption in rat liver mitochondria by indole (circles) and 4 F-indole (squares). The mean and the standard deviation are calculated from four independent repeats.

The effect of indole and 4 F-indole on the rate of oxygen consumption by isolated rat liver mitochondria was measured in the presence of succinate as substrate, but in the absence of ADP in order to preclude any proton movement through the ATP-synthase (Figure 4). Below 0.4 mm, indole does not affect the mitochondrial respiration rate, but when the concentration is raised from 0.4 mm to 0.8 mm there is a 60% increase. In contrast, 4 F-indole affects the mitochondrial oxygen consumption rate at a concentration as low as 0.125 mm. An increase of the 4 F-indole concentration to 0.375 mm raises the rate of mitochondrial oxygen consumption by more than 65%. Thus, in agreement with the electrophysiology measurements, charge transport across the inner membrane of mitochondria is modified in the presence of indole or 4 F-indole. Both molecules uncouple mitochondrial oxidative phosphorylation, with 4 F-indole being the more effective uncoupler, as expected from our in vitro characterization.

Our measurements on diphytanoyl phosphatidylcholine (DPhPC) lipids together with our experiments on mitochondrial oxygen consumption indicate that indole not only affects bacteria, but has the capacity to affect nearby cells that may not themselves produce indole. By its uncoupling action the bacterial indole production could regulate the energy production of surrounding cells. This could give the indole producer an advantage when struggling to survive (e.g. slowing down the energy production in macrophages which are actively fighting against bacteria). Therefore, our data provide a biophysical explanation for how indole may link to the metabolism of bacterial and eukaryotic cells and thus act as an inter-kingdom signal.

## 3. Conclusion

We have shown that indole is a protonophore and that the ionic conductance displays a quadratic dependence on its concentration in solution. Our results demonstrate that the transport of indole is selective and that the charge transport across the lipid membrane is enhanced by an increase in the bathing pH. The efficiency of the indole molecule as a protonophore was increased by the introduction of a fluorine atom in its chemical structure, namely 4 F-indole. These results suggest a biophysical mechanism for small organic molecules, such as indole that might be employed by bacteria to influence the energy production and thus the behaviour of cells in surrounding tissues. It is interesting to note that in the human gut indole concentrations reach concentrations of up to 1 mm[36,37] and thus, mitochondria in cells of the gut epithelium could be directly affected by the bacterial indole production. Our measurements confirm the action of indole and analogues as uncouplers of oxidative phosphorylation, reemphasize the potency of indole in biological systems, and provide a biophysical explanation for indole effects on eukaroyotic cells.

## Experimental Section

Classical electrophysiology was used to study the permeability of lipid membranes to ions in the presence of indole. A lipid bilayer is formed in the round aperture of a Teflon foil (70–90 μm diameter, 25 μm thickness), using the Montal–Mueller technique.[16,17] The setup is shown in the Supporting Information Figure S1. A 2.5% (by volume) hexadecane solution (Sigma Aldrich) was used for coating the Teflon foil before bilayer formation. 5 μl of a solution of diphytanoyl phosphatidylcholine (DPhPC; Avanti Polar Lipids, 5 mg mL^−1^ in pentane) was spread on the water surface in the compartments surrounding the lipid bilayer and subsequently the lipid bilayer was formed. A model 2400, AM Systems, amplifier in the voltage-clamp mode was used for applying the trans-membrane voltage and for measuring the electric current. Electrical noise was screened from the lipid membrane with a Faraday cage connected to the ground of the amplifier. Ag/AgCl electrodes provided the electrical connection between the solutions and the head stage of the amplifier. The ground and the live electrode of the amplifier defined the CIS and the TRANS sides of the lipid bilayer, respectively. The applied voltage across the lipid bilayer was varied in steps of 10 mV between +50 and −50 mV, and for each applied voltage the current was recorded for 10 s (Figure S2). The data acquisition was made with a DAQ card (National Instruments) and a custom written Lab View code. A Gaussian fit was made on the histogram of the current trace and the average value of the current was determined. A linear fit was applied to the current-versus-voltage (I–V) characteristic (±50 mV) and the ionic conductance through the bilayer was extracted from the gradient of the linear fit. The capacitance of the setup containing the lipid membrane, evaluated each time before the measurement of the I–V characteristic, was determined by applying a triangular potential waveform to the lipid bilayer and measuring the resulting phase-shifted square wave. In order to evaluate the capacitance of the lipid membrane only, the capacitance of the setup embedding the lipid membrane was subtracted from the total capacitance of the setup. Fluctuations of the membrane conductance due to variations in the lipid membrane areas,[18] thickness, or composition were taken into account by defining the specific membrane conductance. This was the measured membrane conductance divided by the capacitance of the lipid bilayer. The specific membrane conductance was used in all our figures (specific conductance). Indole (99.9% purity, catalogue nr. 442619, Sigma Aldrich) was first diluted to 125 mm in absolute ethanol (Fisher Scientific) and then diluted to 10 mm in aqueous solution. This solution was used to adjust the indole concentration in the solution surrounding the lipid membrane. A similar procedure was followed for 4 F-indole (97% purity, catalogue nr. 457396, Sigma Aldrich). All measurements were made in 100 mm KCl. The pH of the solutions was controlled, when not otherwise mentioned, using 15 mm potassium phosphate (PB) in acidic or basic form KH_2_PO_4_/K_2_HPO_4_. 10% solution by mass in water of HCl or KOH was used to adjust the pH, when a pH gradient across the lipid bilayer was introduced or when the pH dependence of the membrane conductance was measured.

Mitochondria were isolated from the liver of male Wistar rats using differential centrifugation. Briefly, rats were euthanized with a rising concentration of CO_2_, after which livers were quickly excised and placed in ice-cold MSM buffer (220 mm mannitol, 70 mm sucrose and 5 mm 3-(*N*-morpholino) propanesulfonic acid (MOPS), pH 7.4) plus 2 mm ethylenediaminetetraacetic acid (EDTA). The livers were finely minced and homogenised using a Teflon/glass pestle and mortar. The homogenate was centrifuged at 400 g, to remove nuclei and cell debris, and the resulting supernatant was centrifuged at 7000 g to yield a mitochondrial pellet. The mitochondrial pellet was re-suspended twice in MSM buffer and centrifuged at 7000 g, before being suspended in a final volume of 1 mL MSM. A protein assay was carried out on the final suspension to determine the quantity of mitochondrial protein used in the respiratory assays. Respiration rates of rat liver mitochondria were measured using Clark-type oxygen electrodes (Strathkelvin Instruments Ltd, Glasgow, UK). Respiration rates were recorded and quantified using 782 Oxygen System v4.0 software (Strathkelvin Instruments). Oxygen concentrations were measured in 0.5 mL respiration medium (100 mm KCl, 50 mm MOPS, 1.0 mm KH_2_PO_4_, defatted BSA 1.0 mg mL^−1^, pH 7.4) in two respiration chambers containing magnetic stirrers and maintained at 37 °C. Specific oxygen concentrations were measured initially in the absence of mitochondria, and then following the addition of mitochondria (equivalent of 0.75 mg mitochondrial protein). Mitochondrial Complex II supported respiration was initiated by the addition of 10 mm succinate in the absence of ADP (state II respiration). Succinate was chosen as the substrate in these studies rather than glutamate, as pilot experiments indicated that indole and 4 F-indole inhibited electron transfer to mitochondrial Complex I. Indole or 4 F-indole were titrated into each of the two chambers until a maximum uncoupled respiration rate was observed. After each run, the chambers were thoroughly cleaned with ethanol and water.

## Notes

As a service to our authors and readers, this journal provides supporting information supplied by the authors. Such materials are peer reviewed and may be re-organized for online delivery, but are not copy-edited or typeset. Technical support issues arising from supporting information (other than missing files) should be addressed to the authors.

## References

[1] Lee JH, Lee J (2010). FEMS Microbiol. Rev.

[2] Lacour S, Landini P (2004). J. Bacteriol.

[3] Lelong C, Aguiluz K, Luche S, Kuhn L, Garin J, Rabilloudn T, Geiselmann J (2007). Mol. Cell. Proteomics.

[4] Lee HH, Molla MN, Cantor CR, Collins JJ (2010). Nature.

[5] Chant EL, Summers DK (2007). Mol. Microbiol.

[6] Domka J, Lee J, Wood TK (2006). Appl. Environ. Microbiol.

[7] Hirakawa H, Kodama T, Takumi-Kobayashi A, Honda T, Yamaguchi A (2009). Microbiology.

[8] Bansal T, Alaniz R, Wood T (2010). Proc. Natl. Acad. Sci. USA.

[9] Wikoff WR, Anfora AT, Liu J, Schultz PG, Lesley SA, Peters EC, Siuzdak G (2009). Appl. Environ. Microbiol.

[10] Piñero-Fernandez S, Chimerel C, Keyser UF, Summers DK (2011). J. Bacteriol.

[11] Sakai M, Tohyama K, Mutai M (1982). Int. J. Biochem.

[12] Mitchell P (1961). Nature.

[13] Mitchell P (1966). Biol. Rev. Cambridge Philos. Soc.

[14] McLaughlin SG, Dilger JP (1980). Physiol. Rev.

[15] Chimerel C, Field CM, Piñero-Fernandez S, Keyser UF, Summers DK (2012). Biochim. Biophys. Acta Biomembr.

[16] Montal M, Mueller P (1972). Proc. Natl. Acad. Sci. USA.

[17] Danelon C, Lindemann M, Borin C, Fournier D, Winterhalter M (2004). IEEE Trans. Nanobiosci.

[18] Hanai T, Haydon DA, Taylor J (1965). J. Theor. Biol.

[19] Heytler PG (1980). Pharmacol. Ther.

[20] Borisova MP, Ermishkin LN, Liberman EA, Silberstein AY, Trofimov EM (1974). J. Membr. Biol.

[21] Dilger J, McLaughlin S (1979). J. Membr. Biol.

[22] Cohen FS, Eisenberg M, McLaughlin S (1977). J. Membr. Biol.

[23] Foster M, McLaughlin S (1974). J. Membr. Biol.

[24] Haydon DA, Hladky SB (1972). Q. Rev. Biophys.

[25] Lea EJA, Croghan PC (1969). J. Membr. Biol.

[26] Finkelstein A (1970). Biochim. Biophys. Acta Bioenerg.

[27] Balón M, Carmona C, Muñoz MA, Hidalgo J (1989). Tetrahedron.

[28] Muñoz MA, Guardado P, Hidalgo J, Carmona C, Balón M (1992). Tetrahedron.

[29] Otero N, Mandado M, Mosquera RA (2007). J. Phys. Chem. A.

[30] LeBlanc OH (1971). J. Membr. Biol.

[31] Margin VS, Patushenko LJ, Kristal LJ, Liberman EA, Topaly VP (1969). Biofizika.

[32] Parsegian A (1969). Nature.

[33] Benz R (1978). J. Membr. Biol.

[34] Liberman E, Topaly V (1968). Biochim. Biophys. Acta Biomembr.

[35] Liberman E (1970). Neurosci. Behav. Physiol.

[36] Karlin D, Mastromarino A, Jones R (1985). J. Cancer Res. Clin. Oncol.

[37] Zuccato E, Venturi M, Di Leo G, Colombo L, Bertolo C (1993). Dig. Dis. Sci.

[38] Bansal T, Englert D, Lee J, Hegde M (2007). Infect. Immun.

