# Supplementary material for: The Effect of Bacterial Signal Indole on the Electrical Properties of Lipid Membranes
Source: Chemphyschem. 2013 Jan 9;14(2):417–23. doi: 10.1002/cphc.201200793 (PMC3790958; doi:10.1002/cphc.201200793)
Supplement: Supplementary file 1 [file cphc0014-0417-SD1.pdf]

CHEMPHYSCHEM

Supporting Information

© Copyright Wiley-VCH Verlag GmbH & Co. KGaA, 69451 Weinheim, 2013

The Effect of Bacterial Signal Indole on the Electrical Properties of Lipid Membranes

Catalin Chimerel,[a] Andrew J. Murray,[b] Enno R. Oldewurtel,[a] David K. Summers,[c] and Ulrich F. Keyser*[a]

cphc_201200793_sm_miscellaneous_information.pdf

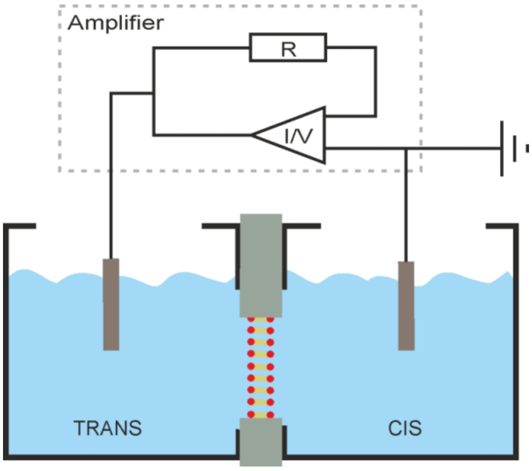

Suplementary Figure 1. Scheme of the electrophysiology set-up used to measure currents through lipid membranes.

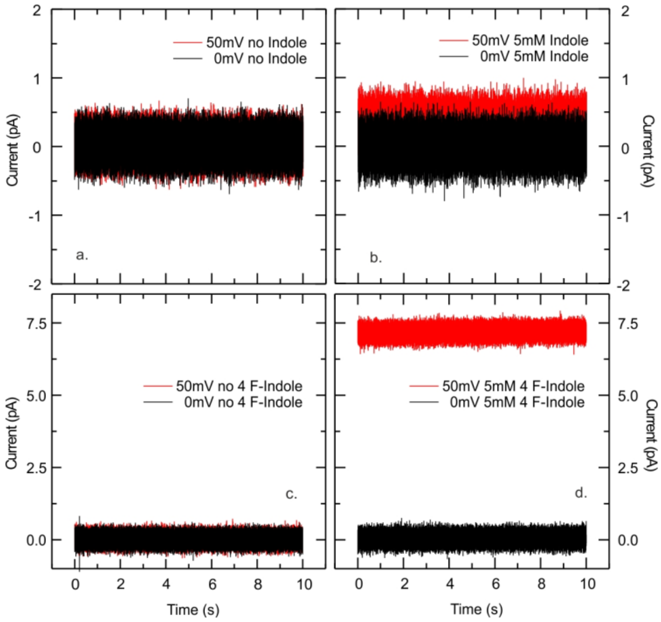

Supplementary Figure 2. Curent traces measured in the presence of indole and 4 F-indole. In the top panells the current through the lipid membrane at 0 mV and +50 mV is measured in the absence of indole, a., and in the presence of 5 mM indole, b. In the bottom panells the current through the lipid membrane at 0 mV amd +50 mV is measured in the absence of 4 F-indole, c., and in the presence of 5 mM 4 F-indole, d. All traces were measured with an aquisiton frequency of 10kHz and a sampling frequency of 50kHz. Post aquisition the traces were filtered with low pass filter with a cutoff frequency of 2kHz.

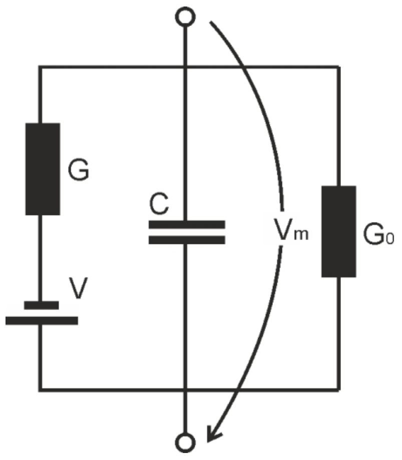

Suplementary Figure 3. Equivalent circuit of a lipid membrane exposed to a pH gradient in the presence of an ionophore which selectively transports charge across the membrane. The capacitance of the lipid membrane is denoted with C. All the other physical measures are defined as in equations 1 to 6.

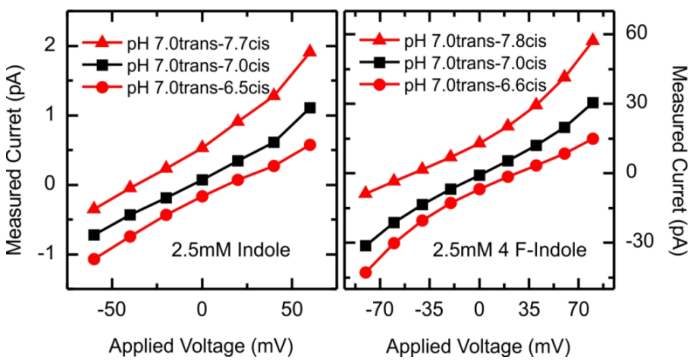

Suplementary Figure 4. The effect of a pH gradient on the IV characteristics of a *DPhPC* lipid membrane in the presence of 2.5 mM indole or 2.5 mM 4 F–indole. The *TRANS* chamber is held at pH 7.0 while the pH in the CIS chamber is varied. Symbols denote the pH on either side of the membrane.
